# Detection of *Echinococcus granulosus sensu lato* microRNAs in cystic echinococcosis patients: An exploratory study using quantitative PCR and digital PCR

**DOI:** 10.1371/journal.pntd.0013833

**Published:** 2025-12-15

**Authors:** Chiara Stocchero, Tommaso Manciulli, Alessandro Zanon, Gaspare Salvi, Susanna A. Sechi, Alessia Pea, Alessandra Cafiso, Paola Pepe, Ambra Vola, Marcela A. Cucher, Enrico Brunetti, Cristina Lecchi, Chiara Bazzocchi

**Affiliations:** 1 Department of Clinical, Surgical, Diagnostic and Pediatric Sciences, University of Pavia, Pavia, Italy; 2 Department of Veterinary Medicine and Animal Science, University of Milan, Lodi, Italy; 3 WHO Collaborating Center for the Clinical Management of Cystic Echinococcosis, University of Pavia, Pavia, Italy; 4 Department of Experimental Medicine, University of Florence, Florence, Italy; 5 Department of Veterinary Medicine and Animal Production, University of Naples Federico II, Naples, Italy; 6 Microbiology and Virology Unit, IRCCS San Matteo Hospital Foundation, Pavia, Italy; 7 Department of Microbiology, School of Medicine, University of Buenos Aires, Buenos Aires, Argentina; 8 Institute of Research on Microbiology and Medical Parasitology (IMPaM, UBA-CONICET), University of Buenos Aires, Buenos Aires, Argentina; 9 Division of Infectious Diseases I, IRCCS San Matteo Hospital Foundation, Pavia, Italy; 10 MOOVET S.r.l., Precision Diagnostics LAB for Animal Health – Spin-off of the University of Milan, hosted at the Department of Veterinary Medicine and Animal Sciences, Lodi, Italy; Cyprus International University: Uluslararasi Kibris Universitesi, CYPRUS

## Abstract

**Background:**

Cystic echinococcosis (CE) is a zoonosis caused by the larval stage of *Echinococcus granulosus sensu lato* (*s.l.).* The adult parasite typically resides in the intestine of canids, while the larval stage, a fluid-filled cyst, primarily resides in the liver and lungs of ungulates and humans. The diagnosis of abdominal CE in humans is mainly based on ultrasound (US) complemented by serology, but both techniques present limitations. Therefore, new diagnostic methods are needed. MicroRNAs (miRNAs) are potential suitable biomarkers for parasitic diseases, as well as for the diagnosis and cyst staging of CE. The objective of this study was to evaluate the presence of three *E. granulosus s.l.* miRNAs (egr-let-7-5p, egr-miR-10a-5p, and egr-miR-71-5p) in the serum of CE patients using novel TaqMan-based quantitative PCR (qPCR) and digital PCR (dPCR) assays.

**Methodology/Principal findings:**

Serum samples from 25 patients with CE, 10 patients with non-CE hepatic lesions, and 10 patients with other parasitic infections were collected. In addition, four cyst fluids were also obtained. Total small RNAs were extracted, reverse-transcribed into cDNA, and amplified.

*Echinococcus granulosus s.l.* miRNAs were detected by qPCR and dPCR in cyst fluid samples, and by dPCR in serum samples. In detail, egr-let-7-5p, egr-miR-10a-5p, and egr-miR-71-5p were amplified in 52%, 48%, and 36% of CE samples, respectively (range: 0.20- 2.86 copies/µL). No egr-miRNAs were detected in patients with non-CE hepatic lesions, whereas egr-miR-71-5p was amplified in only one *Schistosoma* spp. patient. In association with US for CE diagnosis, dPCR assay showed the highest performance when a single *E. granulosus s.l.* miRNA was amplified, achieving a sensitivity of 84% (95% CI 63.9–95.5) and specificity of 95% (95% CI 75.0–99.9). Increasing the number of positive miRNAs required for a positive result reduced sensitivity substantially (40% with two miRNAs, 95% CI 21.1–61.3; 12% with three miRNAs, 95% CI 2.5–31.2).

**Conclusions:**

In conclusion, although this study’s explorative nature and the limited sample size, the detection of *E. granulosus s.l.* miRNAs in the serum of CE patients proved feasible, potentially supporting the medical decision-making process in association with US for CE diagnosis.

## Author summary

Cystic echinococcosis (CE) is a parasitic disease caused by *Echinococcus granulosus s.l.* The adult parasite typically resides in the intestine of canids, while the larval stage, a fluid-filled cyst, primarily resides in the liver and lungs of intermediate hosts (ungulates and humans). The diagnosis of abdominal CE mainly relies on ultrasound (US) and serological tests. However, the former is operator-dependent and the latter is not standardized. This study evaluated the presence of three *E. granulosus s.l.* microRNAs (miRNAs; small non-coding RNA molecules that regulate gene expression) in serum samples of CE patients as possible new diagnostic biomarkers.

Serum from patients affected by CE, non-CE hepatic lesions, and other parasitosis were analyzed using quantitative (qPCR) and digital PCR (dPCR) techniques. At least one of the three miRNAs was detected in 84% of CE serum samples, suggesting dPCR as a possible tool for CE diagnosis, complementary to US in the presence of a suspicious lesion.

## Introduction

Cystic echinococcosis (CE) is a zoonotic disease caused by the larval stage of the flatworm *Echinococcus granulosus sensu lato* (*E. granulosus s.l.*; [1]). The adult parasite resides in the intestine of the definitive host, typically canids, while the larval stage, a fluid-filled cyst also known as metacestode or hydatid cyst, primarily resides in the liver and lungs of intermediate hosts (ungulates and humans), although any organ can be affected. Since humans do not contribute to the perpetuation of the parasite’s life cycle, they are considered to be accidental intermediate hosts [2]. Transmission from the definitive to the intermediate host occurs via the fecal-oral route through the ingestion of parasite eggs, while definitive hosts become infected by consuming parasite larvae (protoscoleces; PSCs), typically through the ingestion of infected offal from livestock or wild game [3]. CE is endemic in areas such as Peru, Chile, Argentina, Uruguay, southern Brazil, the Mediterranean region, central Asia, western China, and East Africa [4], representing a significant public health issue. The global burden of this infection exceeds 1 million Disability-Adjusted Life Years (DALYs), resulting in an annual estimated cost of $760 million [5]. Furthermore, these data may be underestimated due to the high number of asymptomatic patients and the absence of mandatory reporting in many countries [6]. When symptoms occur, they are not pathognomonic and can vary depending on the viability, location, and size of the cyst. Additionally, complications may arise due to cyst rupture or secondary infections [7]. Currently, abdominal CE diagnosis relies primarily on imaging techniques, particularly ultrasound (US), enabling the operator to assess cyst viability and its classification as active (stages CE1-CE2-CE3a-CE3b) or inactive (stages CE4-CE5), according to the current WHO/IWGE classification (World Health Organization Informal Working Group on Echinococcosis; [8, 9]). Serological methods are also used for CE diagnosis to support imaging and clinical data. However, these approaches lack standardization and may yield false negative results, particularly for inactive cysts. False positives can also occur, especially in the presence of other parasitic infections or due to environmental exposure to parasitic eggs [10]. Thus, the identification of additional biological markers is needed to improve CE diagnosis [11,12].

MicroRNAs (miRNAs) are small non-coding RNA molecules that play a critical role in post-transcriptional gene regulation, influencing a wide range of developmental and physiological processes. miRNAs are also actively secreted by cells and can be detected in body fluids [13]. Their stability, ease of isolation from tissues and body fluids, and regulatory role have highlighted them as potential biomarkers for various pathological conditions, including cancer, metabolic disorders, cardiovascular and infectious diseases both in human [14,15] and veterinary [16–18] medicine. In parasitic diseases, host and parasite miRNAs regulate disease progression, with host miRNAs aiding parasite elimination and parasite miRNAs promoting its survival [19,20]. Some studies indicate that in patients with CE at different stages, host miRNAs undergo either upregulation or downregulation [21–23]. However, parasite-derived miRNAs could be considered more reliable biomarkers than host miRNAs, whose levels in serum are also influenced by different cellular sources and unrelated factors [24]. In addition, some miRNAs released by parasites show highly divergent sequences compared to those produced by the host [25,26], representing potential species-specific biomarkers.

In recent years, miRNAs of *E. granulosus s.l.* metacestode have been extensively analyzed. In particular, miRNAs sequencing has been performed on swine cyst wall and PSCs, on sheep liver hydatid cysts [27–29], on exosome-like vesicles (ELVs) isolated from hydatid fluid (HF) of fertile sheep cysts, and on culture medium of *in vitro* PSCs cultures [30]. Notably, five miRNAs (egr-miR-71-5p, egr-let-7-5p, egr-miR-10a-5p, egr-miR-2a-3p, and egr-miR-277a-3p) are consistently expressed across all investigated matrices [24]. In addition, the circulation of some of the most highly expressed *E. granulosus s.l.* miRNAs (egr-miR-2a-3p; egr-let-7-5p; egr-miR-9-5p; egr-miR-71-5p) was demonstrated in the serum and plasma of CE human patients, with alterations in their expression levels observed before and after hydatid cyst removal [31,32], as well as in relation to the presence of different cystic stages [33].

Based on these findings, this study aimed to evaluate the expression of egr-miR-10a-5p in the serum of CE patients for the first time. In addition, egr-let-7-5p and egr-miR-71-5p were evaluated as potential biomarkers for the diagnosis and cyst staging in CE patients, using a specific TaqMan probe assay applying quantitative and digital PCRs as amplification methods.

## Methods

### Ethics statement

The retrospective study was performed according to the guidelines of the Institutional Review Board of San Matteo Hospital Foundation (Pavia, Italy) on the use of biological specimens for scientific purposes in keeping with Italian Law (art.13 D.L gs 196/2003) and was approved by the local Ethics Committee “Lombardia 6” under protocol number 0002311/25 on 19/12/2024. Consent was waived due to the nature of the study and the use of anonymized data, in accordance with institutional policies and in keeping with Italian law.

### Patients, sample collection, and preparation

Samples were obtained from the IRCCS Policlinico San Matteo Hospital Foundation (Pavia, Italy). Twenty-nine samples were collected from CE patients and grouped as follows: sera from CE patients with active, inactive, or both active and inactive cysts (n = 25); HFs from active cysts (n = 4). The diagnosis of CE and the cystic stage were determined during routine US analysis. Serum from patients with no parasitic lesions (NPL; n = 10) and patients with other parasitic infections (OPI; *Schistosoma* spp. n = 6, *Strongyloides* spp. n = 4) were randomly selected and included in the analysis. The diagnosis of NPL was based on the combination of clinical, serological, and imaging data, while OPI was diagnosed through serological testing for *Schistosoma* spp. (LDBIO Diagnostics, Lyon, France) and *Strongyloides* spp. (Bordier products, Crissier, Switzerland) detection. Samples were stored at -80°C for up to ten years until small RNAs extraction was performed. Long-term storage is widely recognized to preserve miRNAs integrity [34].

### Small RNAs extraction and reverse transcription

Total small RNAs were extracted using the Micro RNA Concentrator Kit (A&A Biotechnology, Gdansk, Poland) from 200 µL of each sample (serum or HF). *Caenorhabditis elegans* miRNA cel-miR-39-3p synthetic spike-in (25 fmol final amount; Qiagen, Hilden, Germany) was used as an exogenous extraction control. RNA was quantified using a NanoDrop ND-100 Spectrophotometer (Thermo Scientific, Wilmington, DE, USA) and reverse transcribed into cDNA using the TaqMan Advanced miRNA cDNA Synthesis Kit (Thermo Fisher Scientific, Waltham, MA, USA), following a modified manufacturer’s protocol. Briefly, 3.7 μL of RNA were reverse transcribed, and the miR-Amp reaction cycles were increased to 18 to allow the amplification of low-abundance targets.

### Target selection and evaluation of amplification specificity

*Echinococcus granulosus s.l.* miRNAs (egr-let-7-5p, egr-miR-10a-5p, and egr-miR-71-5p) were selected from the literature based on their abundance in various metacestode matrices and their circulation in the serum of CE patients [24,33,35]. The endogenous human controls hsa-miR-20a-5p and hsa-miR-101-3p were selected to confirm the RNA isolation quality and the absence of possible inhibitors, from sequencing data reported by [21], based on miRNAs that did not show significant differences among healthy subjects and CE patients with active and inactive cysts, with a log_2_ fold change close to zero and the lowest standard error. The synthetic exogenous miRNA cel-miR-39-3p was used to assess small RNAs extraction and reverse transcription efficiency, following the MIQE Guidelines [36]. The TaqMan Advanced miRNA assays (Thermo Fisher Scientific, Waltham, MA, USA) were selected for hsa-miR-20a-5p (Assay ID: 478586_mir), hsa-miR-101-3p (Assay ID: 477863_mir), and cel-miR-39-3p (Assay ID: 478293_mir). Since TaqMan Advanced miRNA assays for egr-let-7-5p, egr-miR-10a-5p, and egr-miR-71-5p were not available, they were custom-designed for research use by ThermoFisher Scientific service. Probes sequences were as follows: TGAGGTAGTGTTTCGAATGTCT-FAM (egr-let-7-5p); CACCCTGTAGACCCGAGTTTGA-FAM (egr-miR-10a-5p); TGAAAGACGATGGTAGTGAGA-VIC (egr-miR-71-5p). To assess the specificity of the custom-designed probes in detecting *E. granulosus s.l.* miRNAs, qPCR was performed on miRNAs extracted and reverse transcribed from HF, as reported below. The PCR products were cloned into the pGEM-T Easy Vector System II (Promega, Madison, WI, USA) as previously described [17]. Plasmid DNA was purified with Wizard *Plus* SV Minipreps DNA Purification System (Promega, Madison, WI, USA), subjected to Sanger sequencing, and sequence specificity was verified through miRbase (https://www.mirbase.org/).

### Quantitative (q)PCR and digital (d)PCR analyses

qPCRs for the amplification of egr-let-7-5p, egr-miR-10a-5p, egr-miR-71-5p, hsa-miR-20a-5p, hsa-miR-101-3p, and cel-miR-39-3p were carried out using the CFX Duet Real-Time PCR System (Bio-Rad, California, USA). Each sample was tested in triplicate, reactions were conducted in a 15-μL final volume containing template cDNA (3 μL), 1X TaqMan Fast Advanced Master Mix (Thermo Fisher Scientific, Waltham, MA, USA), 1X miRNA specific TaqMan Advanced assay, and nuclease-free water to adjust the volume. The thermal cycling conditions were set as follows: 50°C for 2 min, 95°C for 3 min, 45 cycles at 95°C for 15 s and 60°C for 40 s. Fluorescence data were collected at 60ºC. Positive (HF) and no-template controls were added to ensure assay reliability. Samples were considered positive when the amplification curves passed the baseline threshold, manually set at 100 RFU.

The dynamic range of egr-let-7-5p, egr-miR-10a-5p, and egr-miR-71-5p amplifications was determined by performing qPCR on serial dilutions of the purified plasmids, enabling the evaluation of the reaction’s sensitivity in copies/μL of each target.

In addition, to evaluate the circulation of *E. granulosus s.l.*-specific miRNAs in serum of CE patients, a dPCR using QuantStudio Absolute Q Digital PCR System (Thermo Fisher Scientific, Waltham, MA, USA) was performed following the Digital MIQE Guidelines [37]. The reaction was prepared in a final volume of 9 µL, containing 5 µL of template cDNA, 1X Absolute Q DNA Digital PCR Master Mix (Thermo Fisher Scientific, Waltham, MA USA), and 1X miRNA-specific TaqMan Advanced assay. Runs were carried out under the following thermal cycling conditions: 96°C for 10 minutes, followed by 40 cycles at 95°C for 5 s and 60°C for 15 s. Positive (HF) and no-template controls were included. The detection limit of egr-let-7-5p, egr-miR-10a-5p, and egr-miR-71-5p amplifications was determined by performing dPCR on serial dilutions of the purified plasmids, enabling the evaluation of the reaction’s sensitivity in copies of target per microliter of sample (copies/μL). The threshold line was set on the negative control to avoid background noise. In addition, for both qPCR and dPCR reactions, the amount of cDNA, the number of cycles and the thermal profile were carefully optimized to ensure reliable detection of low-abundance targets.

### Statistical analysis

A Principal Component Analysis (PCA) was used for the number, stage, and size of cysts, the treatment (Albendazole - ABZ), and the presence of egr-let-7-5p, egr-miR-10a-5p, and egr-miR-71-5p as an exploratory analysis to detect underlying relationships among variables. Data assumptions were checked, Keiser-Meyer-Olkin (KMO) and Bartlett’s test of sphericity were applied to test the suitability of the data for structure detection. Factor scores were calculated for samples when the component’s Eigenvalue was greater than one, to evaluate the distribution of the subjects according to the considered variables.

The sensitivity and specificity values for the use of miRNAs as an adjunctive test for the diagnosis of CE in the presence of a suspicious lesion were calculated for three scenarios: a sample was considered positive if at least one miRNA, two miRNAs or three miRNAs were identified. ROC curves and AUC values were calculated using STATA 19 SE (STATACorp, USA) for all three scenarios.

## Results

### Demographics and clinical characteristics of patients

According to the WHO-IWGE classification system, 51.7% of CE patients included in the study presented active cysts CE1, CE2, CE3a, CE3b (sera n = 11; HFs n = 4), 20.7% inactive cysts CE4, CE5 (sera n = 6), and 27.6% both active and inactive cysts (sera n = 8). The age of patients ranged between 26–90 years (mean age = 54), and 58.6% of them were female (n = 17). Patients presented a variable number of CE lesions with sizes ranging between 1.2 and 11.8 cm. In detail, 55% of patients had a single cyst (n = 16), 7% two or three cysts (n = 2), 31% four or five cysts (n = 9), and 7% were highly parasitized with more than five cysts (n = 2). Moreover, at the time of sampling, 37.9% of CE patients were undergoing ABZ (n = 11) treatment. Data about CE patients (age, sex, and treatment) and cysts (number, stage, and size) are reported in Table 1. In addition, the age of patients with OPI (*Schistosoma* spp. n = 6, *Strongyloides* spp. n = 4) ranged between 19–74 years (mean age = 39), and 25% of them were female (*Strongyloides* spp. n = 3). Finally, patients with no parasitic lesions (NPL; n = 10) were 4 males and 6 females with the age ranged between 17–83 years (mean age = 59).

**Table 1:** CE patients’ characteristics.

SAMPLE	SEX	AGE	N° CYST	STAGE	SIZE (cm)	ABZ TRM
1CE	M	52	1	CE3b	<5	no
2CE	F	26	1	CE2	5 - 10	yes
3CE	F	75	1	CE4	5 - 10	no
4CE	F	75	1	CE5	5 - 10	no
5CE	F	24	1	CE4	<5	no
6CE	F	64	1	CE5	5 - 10	no
7CE	M	47	1	CE2	<5	no
8CE	F	63	1	CE3a	5 - 10	yes
9CE	F	39	1	CE2	5 - 10	no
10CE	M	48	1	CE2	5 – 10	no
11CE	F	53	1	CE2	5 – 10	no
12CE	F	73	1	CE3a	5 – 10	no
13CE	M	42	1	CE4	<5	no
14CE	M	74	5	CE3b (4) CE4 (1)	>10	no
15CE	M	27	2	CE3a (2)	<5	yes
16CE	F	35	4	CE1 (1) CE3a (2) CE4 (1)	5 - 10	no
17CE	F	64	4	CE4 (3) CE2 (1)	<5	yes
18CE	F	56	5	CE1 (2)	<5	yes
19CE	M	35	4	CE1 (1) CE2 (2) CE3b (1)	5 - 10	yes
20CE	F	57	5	CE3b (4) CE4 (1)	>10	yes
21CE	M	53	>5	CE5	NA	no
22CE	F	90	4	CE3b (2) CE4 (2)	>10	no
23CE	M	57	3	CE3b CE5 (2)	5 - 10	no
24CE	F	52	5	CE1 (1) CE2 (2) CE4 (2)	5 - 10	no
25CE	M	56	>5	CE3b (1) CE4 (1)	NA	no
1HF	F	46	4	CE1 (4)	NA	yes
2HF	F	45	1	CE2	5 - 10	yes
3HF	M	81	1	CE3a	5 - 10	yes
4HF	M	44	1	CE3b	>10	yes

### Extraction and reverse transcription efficiency

Small RNAs were extracted from 45 sera and four HFs (yield range: 7 ng/µL-80 ng/µL). To confirm the RNA isolation quality and integrity, cDNA synthesis and the absence of possible inhibitors, the exogenous (cel-miR-39-3p) and endogenous (hsa-miR-20a-5p and hsa-miR-101-3p) control miRNAs were amplified. Results demonstrated that miRNAs were isolated and reverse-transcribed properly in all samples. In detail, the threshold cycles median of the synthetic spike-in cel-miR-39 was similar in all sample groups (CE, HF, NPL, and OPI). On the contrary, the threshold cycles median of hsa-miR-20a-5p and hsa-miR-101-3p was lower in HFs when compared with human serum samples, confirming the partial isolation of the cyst from the host. qPCR threshold cycles (Cq) of exogenous and endogenous control miRNAs are shown in S1 Fig and S1 Table.

### qPCR and dPCR specificity and sensitivity

Egr-let-7-5p, egr-miR-10a-5p, and egr-miR-71-5p qPCR fragments were amplified and cloned into plasmids, subsequently purified, and subjected to Sanger sequencing. The obtained sequences showed 100% identity with the corresponding miRNAs in miRbase (MIMAT0020227; MIMAT0020236; MIMAT0020237; https://www.mirbase.org/), confirming the specificity of the amplifications. The sensitivity of egr-let-7-5p, egr-miR-10a-5p, and egr-miR-71-5p amplifications in both qPCR and dPCR was defined by testing serial dilutions of the corresponding plasmids. The detection limit of qPCR and dPCR reactions was determined as 10 copies/µL and 0.2 copies/µL, respectively, demonstrating a higher detection sensitivity for the dPCR assay.

### *E. granulosus s.l.* - miRNAs detection and diagnostic performance

**Fig 1:**
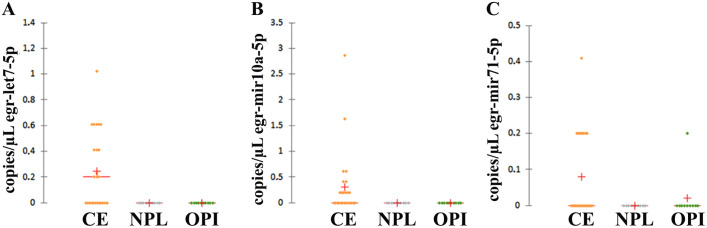
Digital PCR in number of copies/µL of target miRNAs. CE = Cystic echinococcosis; NPL = no parasitic lesions; OPI = other parasitic infections. Red plus= mean value of copies/ µL for each group of patients; red lines = median, defined as the central value of the distribution, with 50% of samples above and 50% below.

The amplification of egr-let-7-5p, egr-miR-10a-5p, and egr-miR-71-5p was performed on all samples with both qPCR and dPCR. The presence of *E. granulosus s.l.* miRNAs in HF samples was confirmed using both assays. Briefly, egr-let-7-5p resulted positive in HF of CE1, CE2 and CE3b patients in both qPCR and dPCR; egr-miR-10a-5p was positive for all HF samples tested in dPCR while qPCR showed positivity only in CE3b; egr-miR-71-5p resulted positive for the four tested samples in both assays (Tables A and B in S2 Table for qPCR and dPCR results, respectively). This finding is consistent with the high abundance of *E. granulosus s.l.* miRNAs in HF, as it represents a parasite-derived matrix. In addition, while qPCR showed no amplification of *E. granulosus s.l.* miRNAs in the serum samples of CE, NPL, and OPI patients, egr-let-7-5p, egr-miR-10a-5p, and egr-miR-71-5p were detected in patients’ serum samples using the dPCR approach (Fig 1 and S3 Table). In detail, egr-let-7-5p was detected in 52% of CE samples (n = 7 with active cysts; n = 2 with inactive cysts and n = 4 with both; copies/µL ranged from 0.20 to 1.02); egr-miR-10a-5p in 48% of CE samples (n = 6 with active cysts; n = 3 with inactive cysts and n = 3 with both; copies/µL ranged from 0.20 to 2.86); and egr-miR-71-5p in 36% of CE samples (n = 4 with active cysts; n = 2 with inactive cysts and n = 3 with both; copies/µL ranged from 0.20 to 0.41). The three target miRNAs were simultaneously detected in 12% of CE samples (n = 3 with active cysts), while no miRNAs were detected in 16% of CE samples (n = 2 with active cysts; n = 1 with inactive cysts and n = 1 with both). Finally, 7 out of 25 patients (n = 5 with active cysts and n = 2 with both active and inactive cysts) were treated with ABZ. No miRNAs amplification was detected only in one ABZ patient with an active cyst. None of *E. granulosus s.l.* miRNAs were detected in NPL, and only one OPI sample showed amplification for egr-miR-71-5p.

The detection of a single miRNA had the highest sensitivity (84%, 95% CI 63.9%-95.5%) and the specificity is 95% (95% CI 75-99.9). Increasing the number of positive miRNAs required for a positive result decreased sensitivity, increasing specificity (S4 Table). Among the five patients with only inactive lesions, four (75%) had at least one positive miRNA. ROC curve analyses confirmed these trends, with the best performing AUC corresponding to the diagnostic test where at least one miRNA was amplified (S4 Table and S2 Fig).

**Fig 2:**
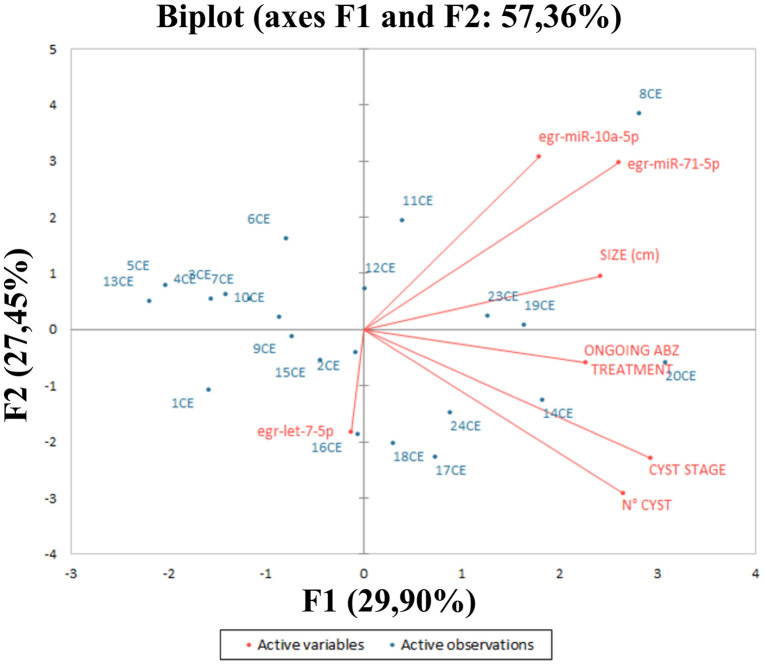
Explorative PCA analysis (KMO = 0.516 and Barlett’s test *P* = 0.011) in 25 CE patients. The correlation circle shows the correlations between the miRNAs, cyst number, size, stage and treatment with ABZ. The PCA identified two principal components with eigenvalues greater than 1, accounting for 57.36% of the total variance (F1-Component 1: Eigenvalue = 2.093, explained variance = 29.90%; F2- Component 2: Eigenvalue = 1.920, explained variance = 27.45%; cumulative variance = 57.36%). Variables with a higher correlation have a smaller degree of separation within the chart; a probable correlation can be predicted if the factors are within 45°. If 2 variables are far from the center and close to each other, they are significantly positively correlated; orthogonal variables are not correlated; and variables on the opposite side of the center line are negatively correlated.

A PCA on CE patients, including the number, stage, and size of cysts, the treatment (ABZ), and egr-let-7-5p, egr-miR-10a-5p, and egr-miR-71-5p copies/μL as variables, was performed (Fig 2 and S3 Table). Acceptable suitability of data for PCA analysis was confirmed (KMO = 0.516 and Barlett’s test *P* = 0.011). The PCA revealed two main factors with Eigenvectors greater than one, which together explain 57.36% of the variation between patients. As shown in Fig 2, the first factor (F1-Component 1; Eigenvalue = 2.093; Explained variance = 29.902%) shows positive loadings for number, stage and size of cysts, the use of ABZ, egr-miR-10a-5p, and egr-miR-71-5p. The second factor (F2-Component 2; Eigenvalue = 1.92; Explained variance = 27.45%; Cumulative explained variance = 57.36%) shows positive loadings for size of cysts, egr-miR-10a-5p, and egr-miR-71-5p.

## Discussion

Current diagnostic techniques for human CE present several limitations, highlighting the need for a novel diagnostic approach. miRNAs have emerged as promising biomarkers for various diseases, including parasitic infections, due to their stability in biofluids and regulatory roles [20,26,38]. In the context of CE, recent studies have investigated miRNAs expressed by different metacestode matrices in swine and sheep [27–30]. In particular, egr-let-7-5p, egr-miR-10a-5p, and egr-miR-71-5p are three of the most highly expressed miRNAs in cyst wall, PSCs, and HF [24], and egr-let-7-5p and egr-miR-71-5p were proposed as potential diagnostic biomarkers and indicators of cystic stage progression in the CE [31,33]. Indeed, the expression of egr-let-7-5p is modulated by the presence of active and inactive cysts [33] while egr-miR-71-5p could be considered a promising biomarker for the early diagnosis of CE and a potential tool for the follow-up of the disease after the removal of the hydatid cyst [31]. Based on these findings, this explorative study investigates the circulation of egr-let-7-5p, egr-miR-10a-5p, and egr-miR-71a-5p in CE patients with active and inactive cysts using a new TaqMan assay that provides reliable information on the specificity and sensitivity of the amplification. The selected miRNAs may play crucial roles in the survival and development of the parasite. Briefly, egr-let-7-5p predicted target genes are associated with longevity-regulating pathways [39]; egr-miR-10a-5p may mediate the host inflammatory response, collagen catabolic processes, and the MAPK cascade during parasitic infection [30]; egr-miR-71-5p may be involved in PSCs development and may regulate macrophage functions during infection [40].

In this work, qPCR results showed the amplification of the three *E. granulosus s.l.* miRNAs in HF samples. Surprisingly, only egr-miR-71-5p was detected in all HF samples, while egr-let-7-5p and egr-miR-10a-5p were amplified only in three (CE1, CE2, and CE3b) and one (CE3b) HF sample, respectively. The higher levels and more frequent detection of egr-miR-71-5p and egr-let-7-5p compared to egr-miR-10a-5p in the metacestode matrix are consistent with existing literature [30]. Moreover, the amplification of egr-miR-10a-5p only in the CE3b HF sample could be attributed to an efficient miRNA extraction yield –confirmed by a lower Cq value of egr-miR-71-5p and egr-let-7-5p– in this sample compared with the other HFs (Table A in S2 Table). CE3b is a stage characterized by denser content, in which collapsed membranes and active daughter vesicles are present [1,41]. This state may enhance the release of cellular parasite components into HF, including miRNAs, in addition to those actively secreted by daughter vesicles, increasing their concentration. In this sense, miRNAs can be secreted either packaged in extracellular vesicles (EVs) or in association with proteins or lipoproteins [13]. In line with this hypothesis, a higher concentration of EVs was found in the inner fluid of senescent metacestodes compared with active parasites in the related species *Echinococcus multilocularis* [25].

Although no amplification of egr*-*miRNAs was detected in serum samples of CE, NPL, and OPI patients using a qPCR approach, dPCR identified a low presence of at least one of the *E. granulosus s.l.* tested miRNAs in 21 out of 25 CE patients and in one out of 10 OPI patients. These findings highlight both the higher sensitivity of dPCR compared to qPCR and the low abundance of circulating egr-miRNAs in human serum, which is below the detection limit of the qPCR reaction, as documented by the sensitivity analyses performed.

The results obtained in this study with qPCR analysis are in contrast with a previous study where both egr-let-7-5p and egr-miR-71-5p were detected in all tested serum samples and considered potential biomarkers for CE [33]. It is important to point out that miRNAs detection in CE patients reported in the literature employed Sybr-Green as fluorescent molecule, which provides high sensitivity but may lead to the amplification of non-specific fragments [42–44]. However, in agreement with the output of this work, negative qPCR results were also reported for egr-miR-71-5p using a hydrolysis probe [25]. In this study, a TaqMan probe-based approach was applied, ensuring high specificity. However, the high specificity of the probe may compromise sensitivity, particularly when the target is present in low copy numbers within the sample. Therefore, dPCR was performed on all serum samples to increase sensitivity, and the limit of detection was defined for both qPCR and dPCR reactions.

Digital PCR results showed amplification of egr-let-7-5p in 14 out of 25 serum samples from CE patients, representing the most frequently identified parasite-derived miRNA tested. The higher circulation of this miRNA in comparison to egr-miR-10a-5p and egr-miR-71-5p in the serum of CE patients could be attributed to its higher expression in ELVs from PSCs and HF [30].

In addition, egr-miR-10a-5p circulation was evaluated and detected in 12 out of 25 serum samples from CE patients. Notably, two samples exhibited higher expression levels of this miRNA compared to the other positive samples. In detail, one sample originated from a patient with CE5-stage cysts, which are classified as inactive clinically [8], although the presence of vital parasitic tissue (including germinal cells) cannot be ruled out using US alone. In fact, a very low chance for reactivation (less than 2%) has been observed in spontaneously inactive cysts in hospital-based studies [45,46]. The other sample was collected from a patient with a CE3a cyst. The structural modifications that characterize this stage may increase the parasite’s interaction with the host, enhancing the immune response and potentially influencing miRNA release into circulation [47].

Finally, the amplification of egr-miR-71-5p was observed in 10 out of 25 serum samples from CE patients and 7 of these also showed amplification for egr-miR-10a-5p. The associated target genes of egr-miR-71-5p and egr-miR-10a-5p are involved in the response to parasitic infection and the positive correlation between the two miRNAs is also supported by the PCA analysis.

In addition, egr-miR-71-5p was detected also in one serum sample collected from a patient with schistosomiasis. This patient underwent abdominal US performed in the context of his workup for schistosomiasis resulting negative for CE. The sequence of miR-71-5p expressed by *E. granulosus s.l.* is identical to *Schistosoma mansoni* sma-miR-71a-5p and *Schistosoma japonicum* sja-miR-71a-5p. The circulation of this miRNA was observed in serum of mice experimentally infected with *Schistosoma* spp. [48,49], and in the serum of human patients with schistosomiasis [50]. These findings highlight a limitation in the specificity of egr-miR-71-5p as a diagnostic marker. Consequently, the detection of miR-71-5p in serum samples of patients without hepatic lesions diagnosed through US examination could not be solely related to CE. Future CE diagnostic panels based on *E. granulosus s.l.* miRNAs should therefore consider the inclusion of additional, species-specific miRNAs, supplementing egr-miR-71-5p, to improve discrimination between CE and other parasitic infections such as schistosomiasis.

Finally, the absence of dPCR amplification for any of the tested egr-miRNAs in four out of 25 serum samples from CE patients could be attributed to their low circulation levels.

The early diagnosis of some diseases (e.g., rheumatoid arthritis and lung adenocarcinoma) or host alteration (e.g., stress conditions) in both human and veterinary medicine is associated with the modulation of miRNAs expression [51–53]. Regarding helminth diseases, some studies demonstrated the presence of parasitic miRNAs in the host’s biological fluids [54,55], even if their role as biomarkers is not yet documented. The biological cycle of different parasites and their interaction with the host may play a crucial role in influencing the circulation of miRNAs released by parasites in the host. In the case of CE, the metacestode is isolated at the site of localization and has limited communication with the host due to the cyst layers [56]. Greater interaction between the metacestode and the host’s immune system may occur when a high number of large, active cysts are present or following cyst rupture [57,58].

In this work, no significant differences in the circulation of egr-let-7-5p, egr-miR10a-5p and egr-miR-71-5p in samples obtained from patients with multiple cysts were observed, but these results do not exclude a modulation of the host’s immune system mediated by egr-miRNAs. In addition, the analysis of the relationship between miRNA detection and cyst dimensions would require more precise and accurate information on cyst size, which is not available in this study. Moreover, no differences in miRNAs circulation were observed in patients undergoing ABZ treatment. However, both these aspects would likely benefit from the analysis of a larger number of samples to be better elucidated. In addition, the PCA outcomes may further improve with the inclusion of a larger patient cohort to better explain the correlation among variables.

In conclusion, this pilot study confirms the circulation of egr-let-7-5p, egr-miR-10a-5p and egr-miR-71-5p in serum samples from CE patients using a novel dPCR approach. However, the limited number of samples analyzed represents a constraint of this study, which should be regarded as a pilot investigation to assess the feasibility of this novel approach for detecting *E. granulosus s.l.* miRNAs in the host. To propose a reliable diagnostic value, the number of samples needs to be increased in future studies. It should be pointed out that the presence of at least one egr-miRNA is not indicative of a CE diagnosis in patients with no abdominal lesions observed through US. Given these limitations, the current scope of the test could only be considered as a complement to US, given its high specificity. Data on the use of serology show that the best assays have a similar performance to those observed for PCR in our study [59]. The method presented in this pilot study is not intended to substitute the use of US as a gold-standard diagnostic tool, but rather to support it as an additional analysis for CE diagnosis. Indeed, while on the one hand the method has the advantage of potentially being less operator-dependent than serology and enables the implementation of standardized protocols [59], on the other hand, the cost of the analyses and the required instrumentation currently prevent its implementation in most endemic areas or its use at a large scale, limiting its applicability to global CE routine diagnosis at present. In addition, the low level of circulating egr-miRNAs is the major pitfall for the clinical implementation of this technique as a fully functioning diagnostic tool. The identification of more abundantly expressed miRNAs in human CE cysts, and their integration into future diagnostic panels, may enhance both the reliability and the diagnostic accuracy of this novel method.

## Supporting information

S1 Fig qPCR threshold cycles of control miRNAs. A = cel-miR-39-3p; B = hsa-miR-20a-5p; C = hsa-miR-101a-3p; CE = Cystic echinococcosis; HF = hydatid fluids; NPL = no parasitic lesions; OPI = other parasitic infections. Red plus= mean; red lines = median.
(TIF)

S2 Fig Assay sensitivity and specificity. ROC curve obtained when a sample was considered positive if at least one miRNA (a), two miRNAs (b) or three miRNAs (c) were amplified.
(TIF)

S1 Table qPCR threshold cycles of exogenous (cel-miR-39-3p) and endogenous (hsa-miR-20a-5p and hsa-miR-101a-3p) control miRNAs. (XLSX)

S2 Table *E. granulosus s.l.* miRNAs in HF samples. A: qPCR results; B: dPCR results.
(XLSX)

S3 Table dPCR results of *E. granulosus* miRNAs (copies/μl). (XLSX)

S4 Table Assay sensitivity and specificity. Sensitivity and specificity values obtained when amplification of one, two or three parasite miRNAs were required for classifying samples as positive.
(XLSX)
