# Supplementary figures and images for: Detection of Echinococcus granulosus sensu lato microRNAs in cystic echinococcosis patients: An exploratory study using quantitative PCR and digital PCR
Source: PLoS Negl Trop Dis. 2025 Dec 15;19(12):e0013833. doi: 10.1371/journal.pntd.0013833 (PMC12721503; doi:10.1371/journal.pntd.0013833)

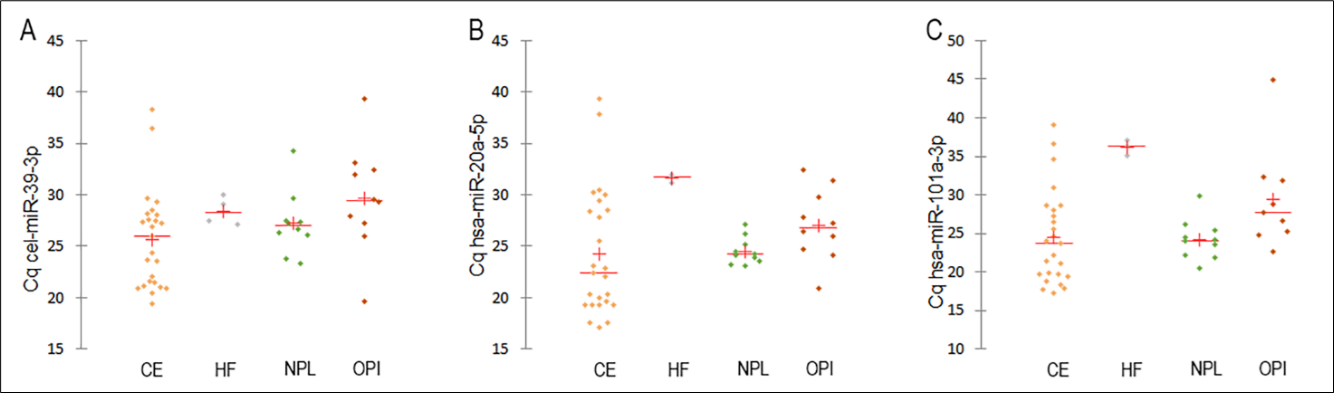

Supplement: S1 Fig — A = cel-miR-39-3p; B = hsa-miR-20a-5p; C = hsa-miR-101a-3p; CE = Cystic echinococcosis; HF = hydatid fluids; NPL = no parasitic lesions; OPI = other parasitic infections. Red plus= mean; red lines = median. (TIF) [file pntd.0013833.s001.tif]

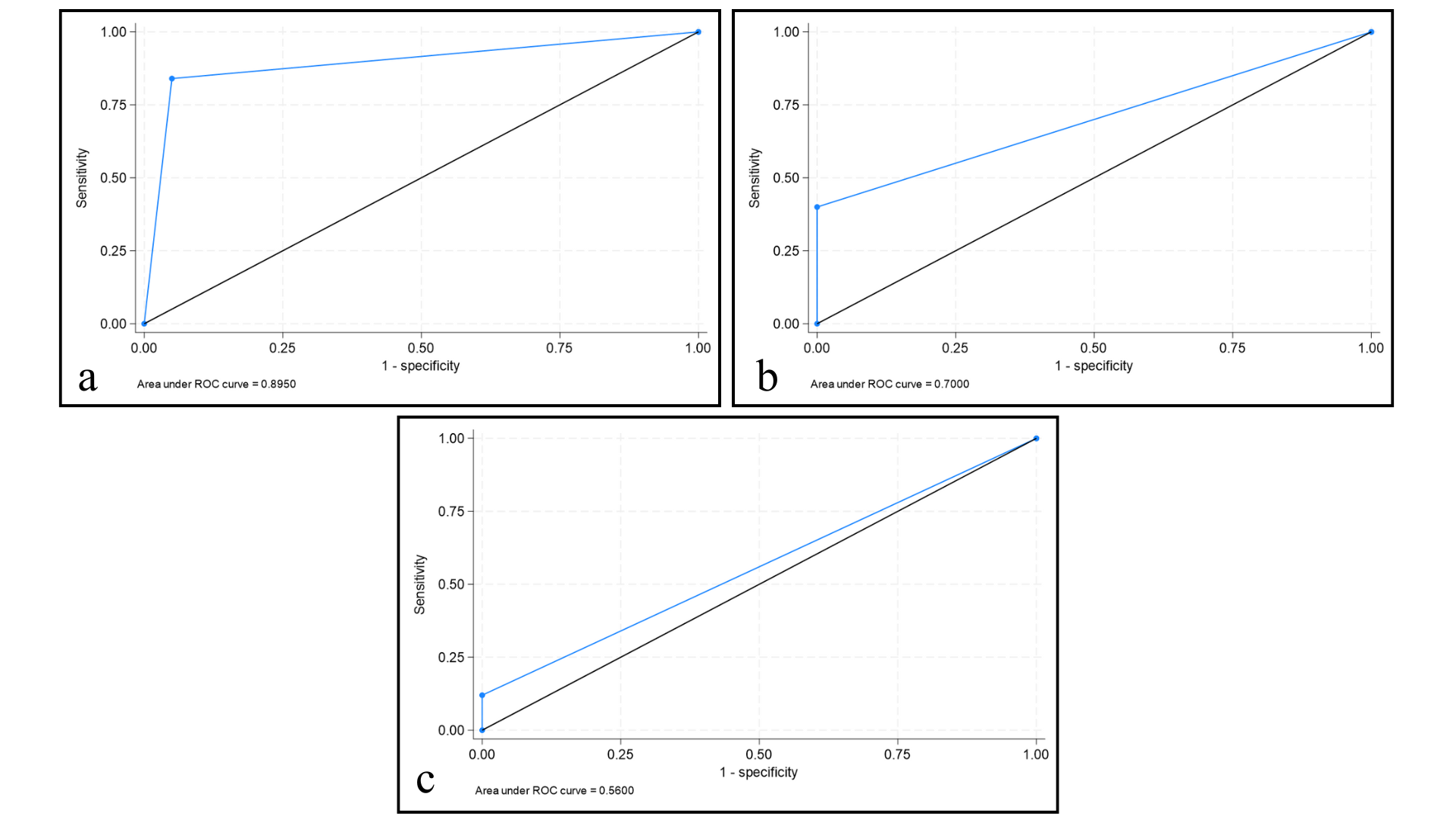

Supplement: S2 Fig — ROC curve obtained when a sample was considered positive if at least one miRNA (a), two miRNAs (b) or three miRNAs (c) were amplified. (TIF) [file pntd.0013833.s002.tif]
